# ISART: A Generic Framework for Searching Books with Social Information

**DOI:** 10.1371/journal.pone.0148479

**Published:** 2016-02-10

**Authors:** Xu-Cheng Yin, Bo-Wen Zhang, Xiao-Ping Cui, Jiao Qu, Bin Geng, Fang Zhou, Li Song, Hong-Wei Hao

**Affiliations:** 1 Department of Computer Science and Technology, School of Computer and Communication Engineering, University of Science and Technology Beijing, Beijing 100083, China; 2 Institute of Automation, Chinese Academy of Sciences, Beijing 100190, China; University of Cape Town, SOUTH AFRICA

## Abstract

Effective book search has been discussed for decades and is still future-proof in areas as diverse as computer science, informatics, e-commerce and even culture and arts. A variety of social information contents (e.g, ratings, tags and reviews) emerge with the huge number of books on the Web, but how they are utilized for searching and finding books is seldom investigated. Here we develop an Integrated Search And Recommendation Technology (IsArt), which breaks new ground by providing a generic framework for searching books with rich social information. IsArt comprises a search engine to rank books with book contents and professional metadata, a Generalized Content-based Filtering model to thereafter rerank books with user-generated social contents, and a learning-to-rank technique to finally combine a wide range of diverse reranking results. Experiments show that this technology permits embedding social information to promote book search effectiveness, and IsArt, by making use of it, has the best performance on CLEF/INEX Social Book Search Evaluation datasets of all 4 years (from 2011 to 2014), compared with some other state-of-the-art methods.

## Introduction

Books are the most widely used archival form of knowledge and entertainment [1, 2]. Nowadays, more and more users are searching books online. Thus, online book searching is an important issue in different academic fields (e.g. library science, informatics and computer science), in economic societies (e.g. e-commerce and social networks) as well as in cultural studies.

With the development of the Internet and social networking services, the information of books is acquired, shared and published in fundamentally different ways. Apart from the professional metadata and contents coming with books, there are also a wealth of user-generated social contents (e.g., ratings, tags and reviews) that come from the Web-described properties, contents and attributes, among the others. For example, the link (http://www.amazon.com/Merchant-Venice-Dover-Thrift-Editions/dp/0486284921) shows a book with both rich professional metadata and user-generated social contents on Amazon.com. Apparently, such social information could help search and find books as people always do on or off line. How to automatically utilize social information and support users in semantic searching books is a widespread critical issue. Though the issue has been mentioned by some (a very small number of) researchers occasionally [2–4], little work has been done to address the question as to how to systematically investigate social information promoting the effectiveness of book search.

It is commonly adopted, by making use of the internal search interface with professional metadata (authors, subjects, publishers, etc.), to search / browse the library website directly (including conventional libraries, e.g., The British Library (www.bl.uk) and National Library of China (www.nlc.gov.cn), and digital libraries, e.g., Open Library (openlibrary.org) and Google Books (books.google.com)) for wanted information. Another conventional way is to use the web search engines (which crawl the publicly available content of digital libraries, e.g., Google (www.google.com) and Baidu (www.baidu.com)) with a user-defined query for books or book-related contents. A lot of research efforts have been conducted over the two conventional ways [5–9].

Alternatively, during book-searching, people show strong interests in social web platforms (e.g., Amazon Books (www.amazon.com/books) and Library Thing (www.librarything.com)), where a wealth of both professional contents of books and rich user-generated social contents (e.g., ratings, tags and reviews) are presented. However, most domain-specific search engines or database retrieval systems for these platforms are mainly based on the books’ metadata and contents, seldom utilizing the user-generated social information. When searching for a book online people usually resort to not only its contents and metadata, but also some book-related comparisons and appraisals from other customers and social networks. Such evaluative information is usually richly embedded in the books on social web platforms. Though there are plenty of researches over information retrieval and data mining for social media [10–12], few researchers have investigated social information for book search.

Does social information help book search? INEX started a track named Social Book Search in 2011. The track aims at investigating book requests and suggestions from the LibraryThing (LT) discussion forums as a way to model book search in a social environment. Several studies have been done along this track. Bellot et al. [13–15] and Bogers et al. [16, 17] focused on the ratings and reviews of books and conducted a weighting function with the number of reviews and ratings for the initial ranking score. Bogers also utilized the ad-hoc reranking method with the tags, ratings and some other information. Considering the importance of context comprehension, Sequential Dependence Model (SDM), a special case of the Markov Random Field model, was also introduced to improve the retrieval performance by introducing sequential consistency. Furthermore, Koolen et al. [3, 18] found that indexing with useful types of social information can help to improve search accuracy. However, due to the complexity of the track, the experimental results indicated that except for these proposed methods, most retrieval models perform no better than the baseline ranking, not as good as in other scenarios due to the complexity of long queries. Only the indexing strategy proposed by Koolen [3] had an appreciable improvement (which means 0.01 on average NDCG@10 over baseline ranking). Although the indexing approach improves the performance of the baseline ranking, the approach ignores the characteristics of social information and processes social information in the same way of professional metadata. Several retrieval models based on initial results optimizing can also combine to make further progress. Therefore, a generic and unified framework for Social Book Search is expected to make use of social information effectively.

How to systematically investigate social information so as to promote book search effectiveness? As described before, book searching can be seen as a searching problem with user-defined queries. To be more practical, the queries have to be much longer than the conventional ones in a freer style and include a large amount of descriptions of the users’ interests. At the same time, searching books with the user’s request and user-generated social contents is more like a task for recommendation systems rather than searching systems. Consequently, searching books with social information can be seen as a search-recommendation hybrid system, i.e., a search system with user-defined queries describes users’ preference or an instant recommendation system without profiles. Here we develop an Integrated Search And Recommendation Technology (IsArt), a generic framework for searching books with rich social information. In IsArt, a search engine is first of all designed to rank books based on book contents and professional metadata. Then, various social features (e.g., ratings, tags and reviews) are extracted and filtered for reranking. For each social feature, a reranking result is obtained with a new recommendation model, a Generalized Content-based Filtering model. Finally, in order to make full use of all reranking results, we use learning-to-rank [19] with Random Forests [20] to adaptively combine a wide range of diverse reranking results with supervised learning.

Generally speaking, the conventional Content-based Filtering model needs a set of preferable items to describe the users’ preferences [21, 22]. In our new Generalized Content-based Filtering (GCF) model of IsArt, on the contrary, a soft preference value is defined for each item ranging from 0 to 1, which can easily catch a wide range of users’ preference. Another novelty is the measure of recommendation in GCF. The conventional Content-based Filtering model takes the sum of similarities with items in the preference set while GCF takes the weighted sum of both the soft preference value and the influence value from the conventional model. This GCF model can adaptively use a soft preference of items, and intelligently merge different recommendation values with a weighted combination. Through the newly proposed GCF model, we introduce the approaches of calculating similarities and design a reranking function utilizing the similarities computing with various social information. In recommendation systems, the purchasing records or the characteristics of interests of users are given as an input, which however are called as the preferences of users in our system. Meanwhile, the queries of search systems also reflect users’ intents and their interests precisely. Comparatively speaking, the queries reflect the users’ instant requests while catalogues mirror their general and long-term intents. In most cases, the instant requests are more appropriate to express the temporal intent. Hence in our framework, we assume that the searching queries act as the input in recommendation systems to represent the characteristics of their interests [23, 24]. Based on this assumption, we design the reranking process as a special case of the recommendation system. This new unified reranking model can easily utilize a variety of rich social information to search and navigate web books semantically.

IsArt is extensively evaluated on all 4 years’ (2011, 2012, 2013 and 2014) datasets of CLEF/INEX Social Book Search Evaluation (https://inex.mmci.uni-saarland.de/tracks/books, now http://social-book-search.humanities.uva.nl/), an international information retrieval evaluation campaign for searching about 2.8 million books with rich user-generated social contents, where IsArt has the best performance (NDCG@10) on all datasets compared with some other state-of-the-art systems.

## Related Work

### State-of-the-art Social Book Search Methods

In the previous Social Book Search Evaluation Campaign and some other studies, several works showed great effectiveness and robustness. Bellot et al. [13, 25, 26] and Bogers et al. [27, 28] conducted several studies on this issue, trying to use social information to promote book search effectiveness. Bellot et al. first analyzed the reviews and ratings of books, computed two relevant scores, and combined these scores with the original ranking result by weighting [13]. Then, they ranked the books based on the “likeliness” which is defined on reviews and ratings. The basic idea is that if a book has a lot of reviews and if its ratings are generally good, then it must be a good book, and could be used to weight the original ranking score [25]. They further revised their book likeliness method, modeled the usefulness of book ratings and tags, and reranked the books with the usefulness score [26]. However, experimental results did not show the benefits of social information. Similarly, Bogers et al., with the help of reviews and book ratings, reranked the books by weighting the original search score [27]. And they further fused ratings, tags and authors for book similarity reranking [28]. After a series of experiments, they found that reranking with the Amazon similar-products performs the best, even better than the strategies of combination. Although the findings provided a way to utilize social information, the results on the testing set urged to conclude that reranking is not a good strategy. In other words, all these techniques are heuristic with specific formulae of using the books’ social information. There is no unified formulation for utilizing a variety of social information. Moreover, no generic framework for searching books with social information exists either. The main reason is that social information that comes from users in the form of reviews, ratings and tags varies widely in length, opinion, clarity and seriousness, as well as in what aspects of the book being discussed. It is challenging to adaptively unify and formulate social information with diverse categories for book search and suggestion [3, 29].

### Related IR and Recommendation Models

Other than the social book search track, there are several similar studies to combine book search with book recommendation, from a different perspective. Kazai et al. developed a social information retrieval model that incorporates different types of social approval votes over documents in a collection, observing that the votes reflecting a broad appeal are most effective [30]. Mooney [31] proposed a Content-based book recommending system that utilizes information extraction and a machine-learning algorithm for text categorization. This approach had the advantage of being able to recommended previously unrated items to users with unique interests as well as to provide explanations for its recommendations. However, for Social Book Search, the above works merely focused on a specific type of contents and were not able to achieve a competitive performance. Koolen [18] investigated the effectiveness of user-generated contents such as tags and reviews, which may contain a large amount of book descriptions not persented in titles, author names or professional subject descriptors. He utilized the Social Book Search dataset to explore the impact of including such user-generated contents in search indexing. Experiments indicated that the indexing strategy with user-generated contents is effective for a large range of information needs. The indexing approach improves the baseline ranking performance by a “procedure-based” strategy. Actually, considering the relationships among books in the search results, there are still varieties of “result-based” methods that can be used to improve the performance.

### Task Description for Social Book Search

LibraryThing.com (LT) is a social book cataloguing website as well as a social platform, where users can post a topic in the forum to seek book recommendations. The requests are described diversely because users have different writing habits and skills. According to the posted topics, some other users may give useful and helpful suggestions for the topic posters.

In the year 2011, Social Book Search was started as a track in the INEX evaluation campaign. Its aim is to investigate book requests and suggestions from LibraryThing (LT) discussion forums as a way to model book search in a social environment. The task assumes that a user who writes a query to a retrieval system, will get a (ranked) list of relevant book records [32]. The retrieval system is expected to order the search results by relevance to the user’s requirements.

**Fig 1:**
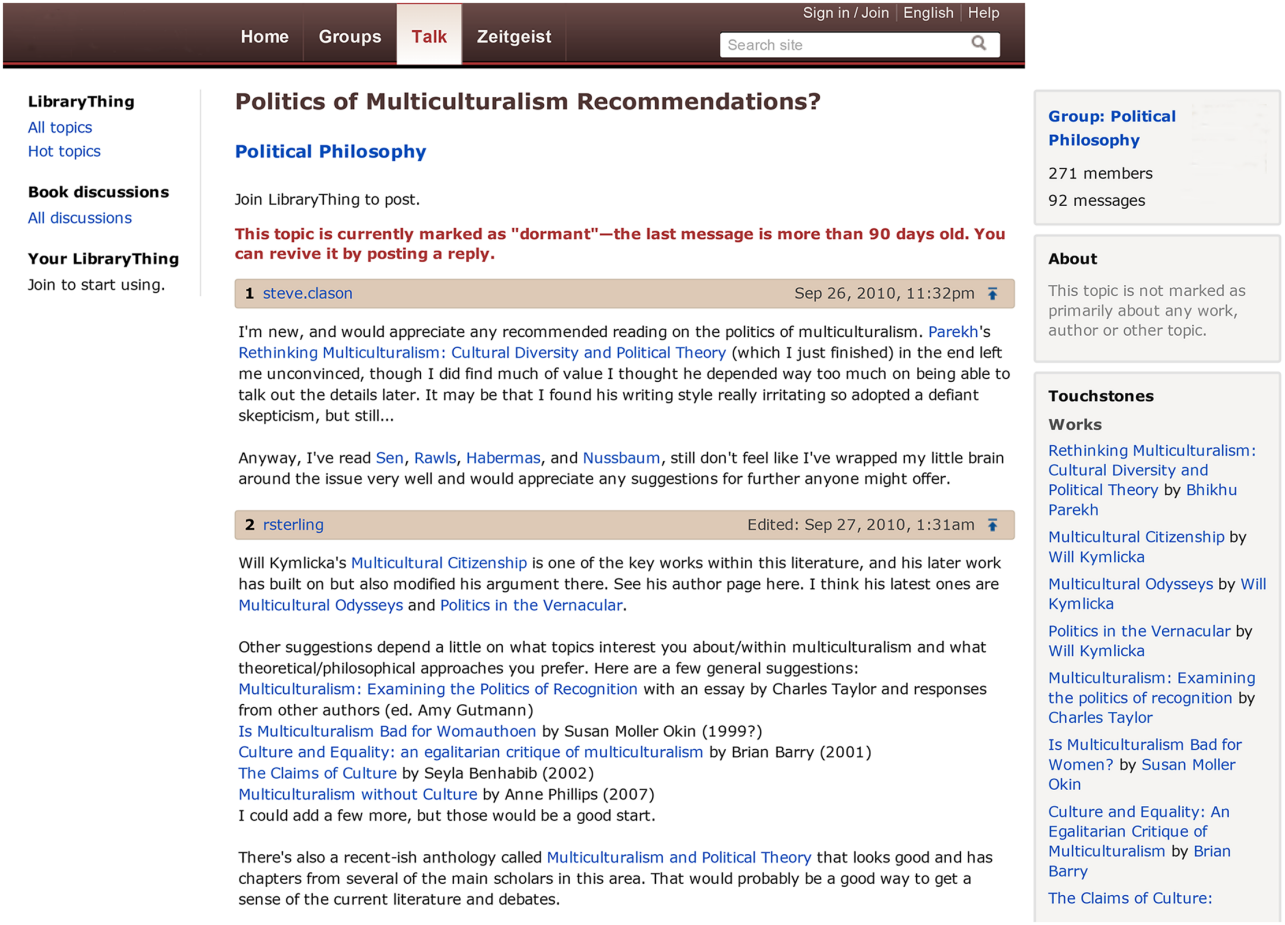
An example topic from LibraryThing.com.

Specifically, the user’s query (i.e., the user’s semantic request, called as a “topic” in LibraryThing.com, examples shown in Fig 1) can not only be a number of keywords, but also one or more book records as positive or negative examples. User requests may vary from asking for books of a particular genre, to looking for books on a particular topic or period or in a certain writing style. The level of details also varies, from a brief statement to detailed descriptions of what the user is looking for. Moreover, the users’ reading profiles in their personal catalogues are partly provided to understand their interests, the list of books they read and their connections with other readers. This catalogue contains the books already read or marked for future reading, and may contain personally assigned tags and ratings. Such preferences and profiles are typical in recommendation tasks, where instead of searching for specific information, the user is looking for suggestions of new items based on his previous preferences and history. The challenge is to develop a method that can cope with such diverse requests.

The topics are selected to construct queries in special forms (and an extra field provided by an annotator from 2013). As is shown in Table 1, each query topic mainly consists of four types of fields: (1) **Title** The 〈title〉 field contains general description headings. Title is given by topic creators, sometimes useless in term of providing suggestions. (2) **Narrative** The first message of recommendation requests, posted by the topic creators, described in the field 〈narrative〉. Narrative is a detailed description of user requests towards the book, with varying lengths and characters. It reflects the user’s diversified information needs, such as writing style, popularity, etc, sometimes also draws some information irrelevant to the requested books. (3) **Group** The 〈group〉 field simply tells the name of the discussion group, which may reveal the nature of the information required, but not necessarily so. And (4) **Query** The 〈query〉 field is provided by an annotator to explain the exact topic in the format of a brief description with more unified length.

**Table 1:** A query XML file with two example topics in INEX Social Book Search.

〈topics〉
〈topic id = “1116”〉
〈query〉introduction book to Lisp〈/query〉
〈title〉Which LISP?〈/title〉
〈group〉Purely Programmers〈/group〉
〈narrative〉It’ll be time for me to…〈/narrative〉
〈/topic〉
〈topic id = “1196”〉
〈query〉books about work for Peace Corps 〈/query〉
〈title〉The Best Peace Corps Novel〈/title〉
〈group〉Returned Peace Corps Readers〈/group〉
〈narrative〉I’m looking for people’s…〈/narrative〉
〈/topic〉
〈/topics〉

The books must be selected from a corpus consisting of a collection with book metadata and social information extracted from Amazon Books and LT, extended with associated records from library catalogues of the Library of Congress and the British Library (see the next section) [32]. The total collection of the documents contains nearly 2.8 million book records. Each book record is represented in a separate XML file with an ISBN number for a unique identification (in Table 2). The collection of books contains a large amount of professional metadata (e.g., 〈title〉 and 〈publisher〉) and wealthy user-generated data (social information, e.g., 〈tags〉 and 〈ratings〉). A name list of all elements in the collection is shown in Table 3. Among these XML elements, several elements are utilizable because of the sparsity. According to incomplete statistics, only a few elements have rich and meaningful textual information. More specifically, only some elements (book, title, isbn) in professional metadata are contained in more than half of the documents in the collection. As for the remaining elements, only *similarproducts*, *tags* (customers tag products based on their interests and suggest a different organization of products), *reviews*, *dewey*, *review*, *rating*, *summary*, *content*, *browseNodes* (a hierarchy of nodes to organize its items for sale), *browseNode* and *tag* are contained in over 1 percent of the documents. The rest of the elements are either sparse or not meaningful/related to contents. As a result, the data is sparse, asking for a search model suitable for sparse data to some extent.

**Table 2:** A document XML file with an example book in INEX Social Book Search.

〈book〉
〈isbn〉0007175000〈/isbn〉
〈title〉Mister Monday〈/title〉
〈manufacturer〉Harper Collins〈/manufacturer〉
〈publisher〉Harper Collins〈/publisher〉
〈dewey〉823.914〈/dewey〉
〈numberofpages〉361〈/numberofpages〉
〈reviews〉〈review〉
〈date〉2003-10-06〈/date〉
〈summary〉So good, you can’t put it down!〈/summary〉
〈content〉Now, I had…〈/content〉
〈rating〉5〈/rating〉〈totalvotes〉7〈/totalvotes〉
〈helpfulvotes〉7〈/helpfulvotes〉
〈/review〉〈/reviews〉
〈tags〉〈tag count = “240”〉fantasy〈/tag〉
〈tag count = “9”〉children’s literature〈/tag〉〈/tags〉
〈similarproducts〉
〈similarproduct〉0439436559〈/similarproduct〉
〈/similarproducts〉
〈browseNodes〉
〈browseNode id = “4”〉Children’s Books〈/browseNode〉
〈/browseNodes〉
…
〈/book〉

**Table 3:** A list of all fields in the document collection in Social Book Search.

book	similarproducts	title
dimensions	tags	edition
reviews	isbn	dewey
editorialreviews	ean	creator
images	binding	review
creators	label	rating
blurbers	listprice	authorid
dedications	manufacturer	totalvotes
epigraphs	numberofpages	helpfulvotes
firstwords	publisher	date
lastwords	height	summary
quotations	width	editorialreview
series	length	content
awards	weight	source
browseNodes	readinglevel	image
characters	releasedate	imageCategories
places	publicationdate	url
subjects	studio	data
imagecategory	name	role
blurber	dedication	epigraph
firstwordsitem	lastwordsitem	quotation
award	browseNode	character
place	subject	similarproduct
tag	seriesitem	

Until the date of writing, the organizers have constructed 4 datasets for this track. There are 211, 96, 386, 680 posted topics collected in INEX 2011, 2012, 2013 and 2014 Social Book Search Task respectively. Along with the posted topics, there are some suggestions given by other users. These suggestions were all collected to construct the ground truth set (composed by manually annotated recommendations) [3] for corresponding topics. To distinguish the importance of these suggestions or recommendations, the relevance values are defined as positive recommendations, negative recommendations, neutral suggestions, and books mentioned for some other reasons to distinguish between books that are mentioned in suggestions. Moreover, the relevance values should be defined to differentiate between recommendations from members who have read the book recommended and those who have not. A suggestion should be assumed as more valuable to the searcher if it comes from someone who has actually read the book. The measure to assign the relevance values of these suggestions is also based on the reactions of the topic creators. Their way of categorizing gold answers are updated almost every year to be more reasonable. Based on the behaviors of the topic creators, a decision tree (shown in Table 4) is built to help to label the relevance values of the suggestions manually, according to the latest version. The evaluation of each year’s tasks is based on the comparison with the official gold answers. For the same topic, because in the variant versions of different years relevant values are defined differently, the gold answers changed a lot.

**Table 4:** Decision Tree for Assigning the Relevance Values.

1 catalogued by topic creator
1.1 pre-catalogued → *rv* = 0
1.2 post-catalogued → *rv* = 8
2 single judgement
2.1 topic creator has read → *rv* = 0
2.2 topic creator has not read positive/netrual/negative → *rv* = 8/2/0
2.3 other member has read positive/netrual/negative → *rv* = 4/2/0
2.4 other member has not read positive/netrual/negative → *rv* = 3/2/0
3 multiple judgements
3.1 multiple has read
3.1.1 some positive, no negative → *rv* = 6
3.1.2 #positive > #negative → *rv* = 4
3.1.3 #positive = #negative → *rv* = 2
3.1.4 #positive < #negative → *rv* = 1
3.1.5 no positive, some negative → *rv* = 0
3.1.6 all neutral → *rv* = 2
3.2 multiple has not read
3.2.1 some positive, no negative → *rv* = 4
3.2.2 #positive > #negative → *rv* = 3
3.2.3 #positive = #negative → *rv* = 2
3.2.4 #positive < #negative → *rv* = 1
3.2.5 no positive, some negative → *rv* = 0
3.2.6 all neutral → *rv* = 2

The track thus combines aspects from retrieval and recommendation [32]. On the one hand, the task is similar to directed information retrieval, with the requirement to search relevant books according to the user’s need described in the forum. On the other hand, users may have particular preferences for writing style, reading level, knowledge level, novelty, unusualness, presence of humorous elements and possibly many other aspects. To some extent, these preferences can be reflected by the user’s reading profile in their personal catalogue.

## Methodology

### Framework of IsArt

**Fig 2:**
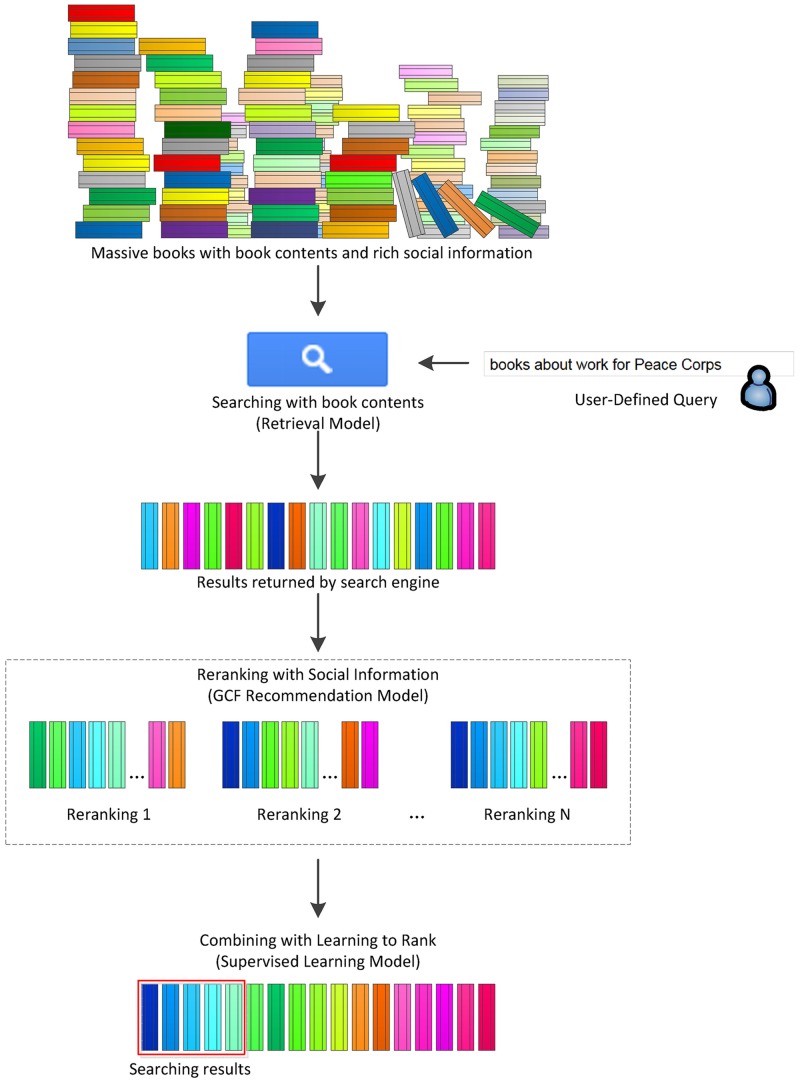
Framework of IsArt, which composes of three major steps: (1) Searching with book contents by retrieval model, (2) reranking with social information by GCF (Generalized Contented-based Filtering) recommendation model, and (3) combining with L2R (Learning-to-Rank) by supervised learning model.

IsArt provides a generic framework for searching and finding books with a variety of rich social information on the Web. Book description in IsArt is mainly shown in Fig 2. However, a universal framework, IsArt, is constructed only on the basis of several common fields (*ratings*, *tags*, *reviews*, *browsenodes* and *similar-products*). These social information features are commonly used in books from social cataloging, e-commerce or SNS websites. There may be more useful information that can be applied into this framework for a specific task such as the Social Book Search Track. In this paper, we discuss the common fields that are only mentioned above. The whole system comprises of three major steps. First, book contents are utilized for book search. Given a user-provided query, the search engine usually searches books based on book contents, professional metadata and other related contents. Then a initial ranking list over books is obtained. The search results depend on the book contents mainly. Second, reranking with social information is conducted. Various social features (e.g., ratings, tags and reviews) are extracted from social-information related documents, and the corresponding feature vectors are constructed. For each feature vector, a reranking result is obtained with the new Generalized Content-based Filtering (GCF) model, which is introduced in the next section in detail. We ignore the catalogue’s influence due to the definition of the ground truth where the relevances of the pre-catalogued products are given as “0”. The reranking results here are mainly based on social information with the recommendation model. Finally, the L2R technique is used to combine all diverse reranking results from social information features. By adaptively and intelligently combining all available knowledge with supervised learning, the final ranking results are achieved on the basis of an integration strategy.

### GCF: Generalized Content-based Filtering

The Content-based Filtering model is a classical but popular model in recommendation systems. The basic idea of this model is to mark the products in the purchasing records or the profiles with “the user like it”, representing the users’ preferences. Items that match the user’s preferences most will be recommended, which also means to find the most similar products to those in the profiles [33]. Though being widely applied, the model has a severe but not obvious pitfall where the difference of levels in preferences is ignored. It is insufficient to mark the products in purchasing records or the profiles with “like” because people always add an adverb, e.g. very, a little, before the word “like/dislike” in their expressions. Another disadvantage of using purchasing records or the profiles to represent the users’ preferences is the cold-start system. In a cold-start system without any purchasing records, it is difficult to learn the preferences of new users [34, 35]. Moreover, the conventional Content-based Filtering model also tends to recommend items that are similar to those they used to like. That means if the preferences change over time, the model may recommend products that become irrelevant [36]. As a result, it is essential to combine the catalogue with instant requests. In the following part, we will extend the model and propose a Generalized Content-based Filtering (GCF) model [29]. GCF can adaptively and flexibly utilize different preferences of the users with a soft weight, rather than only using a set of fixed preferable items. The proposed model includes the following stages:

#### Labeling original preference values

As mentioned above, usually in a recommendation system, the purchasing records or the characteristics of the users’ interests are given to show their preferences. We do not describe the users’ preferences represented merely by purchasing records. Instead, we define an original preference for each product. In a recommendation system, the original preference can usually be labeled by users manually, ranging from 0 to 1. “0” stands for default and the larger the value is, the more preferable the product will be. Conventionally, the products in the catalogue are assigned with “1”, but in our model these preference values vary with specific scenarios. If the users’ preferences are described by their profiles or something else in the form of text, the same to the representation of products, there is also an automatic way to label the empirical preferences by calculating the relevance between the documents and the user’s features with

$$pv\left(d\right)=\begin{array}{c} p\left(r_{i}|d,\mu \right)=\frac{f_{r_{i},d}+\mu p\left(r_{i}|C\right)}{|d|+\mu } \end{array}$$ (1)

where *pv*(*d*) is the preference value of document *d*, *r*_*i*_ is one of the word features of the queries, standing for the users’ interest, *μ* is the Dirichlet smoothing parameter and |*d*| is the length of document *d*. And *f*_*r*_*i*_,*d*_ represents the times that feature *r*_*i*_ appears in document *d*, while *p*(*r*_*i*_|*C*) shows the probability of feature *r*_*i*_ in the set of features, and |*C*| stands for the sum of the frequencies of all words. As a result, *p*(*r*_*i*_|*d*, *μ*) stands for the polynomial distribution estimated with Dirichlet smoothing, which is a classic strategy in the conventional search engine. For simplicity, if the preference is represented by a long description, this description will be regarded as a query (pre-processing by removing stop-words and stemming). Thereafter, all books are labeled with normalization probability scores from the search engine, where the logarithms calculation is not conducted in the last step.

#### Extracting features and calculating similarities

In this stage, the purpose is to accurately calculate the similarities of two books. Obviously a single strategy cannot satisfy the requests. The adoption of more strategies with diverse sources can complement in a better way. Hence, firstly we determine a set of features *F*, according to the structure of the description over documents. For each feature in *F*, a feature vector is extracted from the descriptions. For example, tag *t* in Social Book Search Track is a feature in *F*. In this case, we assume that there are only two different tags in total. The tag vector of product *i* and *j* are $\vec{t_{i}}=\left[3,0\right]$ and $\vec{t_{j}}=\left[0,5\right]$. That means 3 users tag the document *i* with the first tag while 5 users tag document *j* with the second tag. In this way, for a feature *f*, the feature matrix of the whole document collection is constructed as

$$F=\left[\begin{array}{ccc} f_{11} & \cdots & f_{1m}\\ \vdots & \ddots & \vdots \\ f_{n1} & \cdots & f_{nm} \end{array}\right]$$ (2)

where *m* is the total number of distinct features *f*, each line vector in the matrix stands for the feature vector of the corresponding product.

With the matrix, we can calculate the similarity of two products *i* and *j* by computing the similarity of the two feature vectors *f*_*i*_ = [*f*_*i*1_, ⋯, *f*_*im*_] and *f*_*j*_ = [*f*_*j*1_,⋯, *f*_*jm*_]. Similar to the conventional Content-based Filtering model, usually the cosine similarity is chosen,

$$sim_{ij}\left(f\right)=\cos\left\langle \vec{f_{i}},\vec{f_{j}}\right\rangle =\frac{\vec{f_{i}}\cdot \vec{f_{j}}}{|\vec{f_{i}}\left|\right|\vec{f_{j}}|}$$ (3)

where $\vec{f_{i}}\cdot \vec{f_{j}}$ means the dot product of two vectors, and $|\vec{f_{i}}|$ and $|\vec{f_{j}}|$ represent their norms respectively.

3) *Computing Final Recommendation Value*. We define the score calculated from the conventional Content-based Filtering model as the influence value by

$$inv\left(d_{i}\right)=\sum_{j=1}^{N}sim_{ij}\left(f\right)\cdot pv\left(d_{j}\right)\left(j\neq i\right)$$ (4)

Here, a weight is multiplied to the similarities before they are summed together in order to emphasize the different importance of products, which is caused by the preference values.

Considering the effects from both the preference value (*pv*) and the influence value (*inv*), we design a general weighting formulation for combination [28]. The weighted sum of the preference value and the influence value is defined as the final recommendation value (*rv*) with

$$rv\left(d_{i}\right)=\alpha \cdot pv\left(d_{i}\right)+\left(1-\alpha \right)\cdot \frac{1}{N-1}\cdot inv\left(d_{i}\right)$$ (5)

where a normalization coefficient $\frac{1}{N-1}$ is multiplied in order to unify the dimension. Note that only products in the top *N* list of the preference value ((the value *N* depends on the request of accuracy, here we choose *N* = 1000)) are considered in Stage 2) and 3).

Obviously, the proposed GCF model can be applied easily in most recommendation systems for various products, which only differs in feature extraction in terms of various structures of the product descriptions. Moreover, we design a recommendation algorithm with this GCF model (shown in Table 5).

**Table 5:** Algorithm of GCF (Generalized Content-based Filtering) model.

**Input:**
*P*: preference value set, empty set allowed.
*D*: products set.
**Output:**
*R*: set of recommended products.
**Parameter:**
*pv*(*i*): preference value of each product *i*.
*inv*(*i*): Content-based influence value of product *i*.
*rv*(*i*): final recommendation value of product *i*.
*vec*_*i*_, *vec*_*j*_: feature vectors of product i, j.
*sim*_*ij*_: similarity of product i, j.
*N*: threshold to be reranked.
*k*: number of product to be recommended.
**Procedure:**
1: **For** *i* = 1, 2, ⋯, *N*
2: **If** *P* is not empty
3: set *pv*(*i*) = *p*_*i*_default 0
4: **Else**
5: Calculate *pv*(*i*) for product *i* by Eq (1).
6: **End**
7: Choose top N products.
8: **For** *i* = 1, 2, ⋯, *N*; *j* = *i*, *i* + 1,⋯, *N*
9: Extract feature vectors *vec*_*i*_, *vec*_*j*_ of product *i*, *j*
10: Calculate *sim*_*ij*_ by Eq (3) with *vec*_*i*_, *vec*_*j*_.
11: **End**
12: Calculate *rv* for all products by Eq (5).
13: Choose top *k* products added into *R*.
14: Output *R*.

Note that in Stage 1) with GCF, if the preference values of all items in the purchasing records are manually labeled with 1 (otherwise with 0), and *α* = 0, i.e., the preference’s influence is ignored, the procedures and the results of GCF will be the same to the conventional (kNN) Content-based Filtering model. Consequently, the conventional Content-based Filtering model is a special case of our proposed GCF model.

### Implementation of IsArt

In searching with book contents for IsArt, open-source Galago is adopted as the search engine (can be found at http://www.galagosearch.org). All the fields in documents are utilized for indexing and the stop-words are removed from queries during pre-processing. Generally speaking, ranking is one key function in information retrieval [37]. Here, the query likelihood ranking model [38] is adopted, where the probability of the query content appearing in the documents is used to rank the documents with language models. The documents’ priori probabilities are assumed the same, so documents can be ranked by the conditional probability *P*(*Q*|*D*). With Dirichlet smoothing, the estimated probability is calculated with

$$P\left(Q|D\right)=\prod_{i=1}^{n}p\left(q_{i}|D\right)=\prod_{i=1}^{n}\frac{f_{q_{i},D}+\mu \frac{c_{q_{i}}}{\left|C\right|}}{|D|+\mu }$$ (6)

where *f*_*q*_*i*_,*D*_ stands for the amount of times the word/phrase *q*_*i*_ in query *Q* occurs in document *D*, |*C*| and *c*_*q*_*i*__ are the frequency of the query word *q*_*i*_ in all documents respectively, and *μ* is the Dirichlet smoothing parameter, and |*C*| stands for the sum of the frequencies of all words. In this way, documents are scored and ranked by

$$\log P\left(Q|D\right)=\log\prod_{i=1}^{n}p\left(q_{i}|D\right)=\sum_{i=1}^{n}\log\frac{f_{q_{i},D}+\mu \frac{c_{q_{i}}}{\left|C\right|}}{|D|+\mu }$$ (7)

In the reranking process in IsArt, we use a reranking model based on a new recommendation model, the Generalized Content-based Filtering (GCF) model. As described above, the GCF model adaptively uses a soft preference (ranging from 0 to 1) of items, and intelligently merges different recommendation values with a weighted combination. This generic reranking model in IsArt comprises three major stages: 1) labeling the preference values with the scores given by the search engine, 2) extracting features and calculating similarities with social information, and 3) computing the final recommendation values by weighting the preference values and the influence values. Here, a preference value refers to the book’s appeal defined by the description of books and users, and an influence value means the influence of social information on the users’ preferences. In general, there are several common social features for books on the Web (e.g., Amazon.com), i.e., tags, ratings, reviews, similar-products and browse-nodes. In our system, we focus on these five social information related contents during book searching. A *tag* is a collaboratively generated, open-ended labeling system that enables Internet users to categorize books. A *rating* is the user’s evaluation or assessment over books in terms of quality. The *reviews* are varied forms of literary criticism, informal comments, discussions, or just personal feelings in which a book is analyzed based on content, style, merit, and other related issues. A book’s similar products (specifically provided on Amazon.com) contain a list of books marked with similar books by Amazon, where at most 10 books are on the list. The browse nodes, from a hierarchy of nodes, organize their items for sale by Amazon, each node representing a collection of items for sale.

According to these various social features and their combinations, we design 11 different reranking methods: Tag-Rerank (*T*), Node-Rerank (*N*), Item-Rerank (*I*), DeepItem-Rerank (*D*), RatingReview-Rerank (*R*), RatingBayes-Rerank (*B*), Tag-Node-Rerank (*TN*), Item-Tag-Rerank (*IT*), Deep-Tag-Rerank (*DT*), Item-Tag-Node-Rerank (*ITN*), and Deep-Tag-Node-Rerank (*DTN*). Here based on the social contents on Amazon.com, we mainly describe Stage 2) and 3) of the generic reranking model with social information, i.e., extracting features, calculating similarities, and computing the final recommendation value.

Tag-Rerank (*T*) performs the reranking task with 〈tags〉. Specifically, *T* matches the field 〈tag〉, in which the amount of different tags is calculated from the selected books and used as the number of dimensions of the feature vectors. The values of the elements in the vector are extracted from attribute *count*. Then, the similarity *sim*_*ij*_(*f*) of two feature vectors $|\vec{f_{i}}|$ and $|\vec{f_{j}}|$ is calculated by Eq (3).

Node-Rerank (*N*) reranks books with 〈browse-node〉 information, where the amount of corresponding fields is counted as the number of columns, and the values of the elements in the vector are also extracted from attribute *count*. Although 〈browse-node〉 is not social information but controlled metadata, we find that our framework is also suitable for this because it contains some useful content and can be applied to decide the similarities between each two books. In the same way, the similarity between two vectors is also computed by Eq (3).

Item-Rerank (*I*) and DeepItem-Rerank (*D*) (which means two products are regarded as similar if they are similar to the same products through similar-products list) perform reranking with social contents about similar products. These two methods (*I* and *D*) match the field 〈similar-product〉, providing a unique way to calculate similarities between two books. For *I*, we simply think if book *a* is in the top 10 list of book *b*’s <similar-product>, the similarity between these two books is 1, otherwise 0, which is shown by

$$sim_{ij}\left(I\right)=\left\{\begin{array}{cc} 1, & \text{i}\;\text{is}\;\text{in}\;\text{j}’\text{s}\;\text{similar-product}\;\text{collection}\;\text{or}\;\text{vice}\;\text{versa}\\ 0, & \text{else} \end{array}\right.$$ (8)

Considering the transitivity of similarities, *D* is related to similar products with respect to their similar products. We simply define that book *b* and *c* are similar, if book *a* and *b*, and book *a* and *c* both are similar in *I*. We do not utilize a further chain because no change occurs when analyzing with this further chain in most cases. As a result, the similarity for *D* can be calculated by

$$sim_{ij}\left(D\right)=\left\{\begin{array}{cc} 1, & sim_{ij}\left(I\right)=1\;\;\;\;or\\ & \exists \;\;\;\;\;\;k\neq i,\;\;k\neq j,s.t.\;\;sim_{ik}\left(I\right)=sim_{jk}\left(I\right)=1.\\ 0, & \text{else} \end{array}\right.$$ (9)

In IsArt, we further propose several reranking methods by the combining the social information contents with tags (*T*), browse nodes (*N*), and similar products (*I* and *D*). The first group is Tag-Node-Rerank (*TN*), which combines the information of tags and browse-nodes. Directly, *TN* feature matrix is the connection matrix of *T* and *N*. Then, Eq (3) is used to calculate the similarities. The second group is based on utilizing the information of similar products and the information of tags and browse nodes. As is known, most of the similarities for *I* and *D* are 0 because of the upper limit of similar products. The sparsity may have an influence on the performances of the reranking model. Consequently, we propose a series of methods, i.e., *IT* the combination of *I* and *T*), *DT* (the combination of *D* and *T*), *ITN* (the combination of *I* and *TN*), *DTN* (the combination of *D* and *TN*) to solve the problem. Take *IT* as an example, shown in Eq (10). Other combination methods are constructed in the same way.

$$sim_{ij}\left(IT\right)=\left\{\begin{array}{cc} 1, & sim_{ij}\left(I\right)=1.\\ sim_{ij}\left(T\right), & \text{else} \end{array}\right.$$ (10)

After calculating similarities with the above reranking methods (*T*, *N*, *I*, *D*, *TN*, *IT*, *DT*, *ITN* and *DTN*), the influence value is available. The influence value *inv*(*d*_*i*_) is derived from the corresponding preference value (*pv*(*d*_*j*_)) with the similarity by weighting (Eq (4)).

We also integrate the two classical methods (RatingReview-Rerank (*R*) and RatingBayes-Rerank (*B*)) into a reranking model with such social information as reviews and ratings. The newly integrated methods are specifically designed for calculating the influence value in GCF. Among these two methods, *R* is designed according to the idea that if a book has a lot of reviews with good ratings in general, then it must be a high-ranked book. The influence value of this method is calculated by

$$inv\left(d_{i}\right)=log(|reviews\left(d_{i}\right)\left|\right)\times \frac{\sum_{r\in R_{d_{i}}}r}{\left|reviews(d_{i})\right|}\times pv\left(d_{i}\right)$$ (11)

where *R*_*d*_*i*__ is the set of all ratings given by users for book *d*_*i*_, |*reviews*(*d*_*i*_)| is the number of reviews, *pv*(*d*_*i*_) is the preference value of *d*_*i*_, and *pv*(*d*_*i*_) is from “searching with book contents” in IsArt.

In the meantime, the other method *B* focuses on the idea that the average becomes more reliable and less sensitive to outliers, as more users rate the same work [39, 40]. The Bayesian averaging of ratings takes the number of users who have rated the book into consideration. A book’s *BA* score is calculated by [41]

$$BA\left(d_{i}\right)=\frac{\hat{n}\cdot \hat{m}+\sum_{r\in R_{d_{i}}}r}{n+\hat{n}}$$ (12)

where *R*_*d*_*i*__ is the set of ratings for *d*_*i*_, $\hat{m}$ is the average unweighed rating and $\hat{n}$ is the average number of ratings over all the books in the top *N* list. With the Bayesian average rating, the influence value is computed with

$$inv\left(d_{i}\right)=\frac{1+BA(d_{i})}{1+BA_{max}}\times pv\left(d_{i}\right)$$ (13)

Lastly, following the GCF model, for all reranking methods (*T*, *N*, *I*, *D*, *R*, *B*, *TN*, *IT*, *DT*, *ITN* and *DTN*), we compute the final recommendation value (*rv*(*d*_*i*_)) available through Eq (5).

In combining with L2R of IsArt, we use a L2R technique to combine various reranking results from diverse social information contents. As described above, in searching books with social information, several sub-reranking strategies with different social features are investigated. Generally speaking, these sub-strategies are complementary, thus should be merged. However, in most conventional systems, the combination and the parameter adjustments are usually based on manual or semi-manual rules, which are time-consuming and error-prone. Learning-to-Rank (L2R), a supervised learning method, is able to solve such problems [42]. First, data is labeled for preparing the training data. Then the features, here the reranking results with social information, are extracted to represent the document. Afterwards, a learning model is chosen to learn the training data. Finally, the documents are scored with the learned model. In IsArt, after reranking with social information, 11 different reranking results are achieved. An L2R tool (RankLib (people.cs.umass.edu/~vdang/ranklib.html)) with Random Forests then will be used to combine the 11 reranking results. Thus, the learner’s parameters are optimized and selected with the K-fold (here we use *K* = 10) cross-validation on the training set.

## Results

Here, we first describe experimental results about the effectiveness of our proposed framework. The topics of INEX 2013 are utilized to compare the reranking results with the initial ranking results with all query fields and all document fields indexed as the baseline. Then the method *T* is analyzed in detail to find out why the reranking process is effective. To show the necessity of all reranking methods, we compare the runs of different methods coming from a similar source. Afterwards, we compare our results with other top competitors of INEX Social Book Search (from 2011 to 2014) as control groups. In 2011, the book collections do not contain controlled metadata, according to the overview papers of that year. Hence, we remove controlled metadata and do not use the methods *N*, *TN*, *ITN* and *DTN* (because these methods involve controlled metadata Browsenodes) in 2011 to be fair. Moreover, according to the workshop papers, the competitors trained the parameters in the former track. Hence, our system is trained in a same way (to be fair for comparison). The research on the INEX Social Book Search datasets has been approved by the organizers with the license agreement.

### Experimental Setup

In Amazon XML collections, 19 XML fields are selected for book representations. In our experiments, we did not distinguish the influence of different fields’ types. We simply indexed all of available XML fields, including professional metadata, controlled metadata and the social contents. Moreover, there are 4 major fields contained in a topic, 〈*title*〉, 〈*narrative*〉, 〈*query*〉 and 〈*group*〉. We removed the stop words and stemmed all words with Porter Stemmer. All these four fields were used as the queries. Specifically, for SBS 2011 and 2012 datasets, there is no 〈*query*〉 field, so we used other three fields while for SBS 2014 dataset, the queries were enriched with personal catalogues, which was ignored in our experiments. We used the *batch—search* command in *Galago* toolkit for initial ranking, which embeds Dirichlet Smoothing with the query likelihood model (shown in Eq (7)). Dirichlet Smoothing parameter *μ* is set to default 2000 (different than other open-source search engine like *Indri* which set *μ* = 500).

### Effectiveness comparisons of different components in IsArt

**Fig 3:**
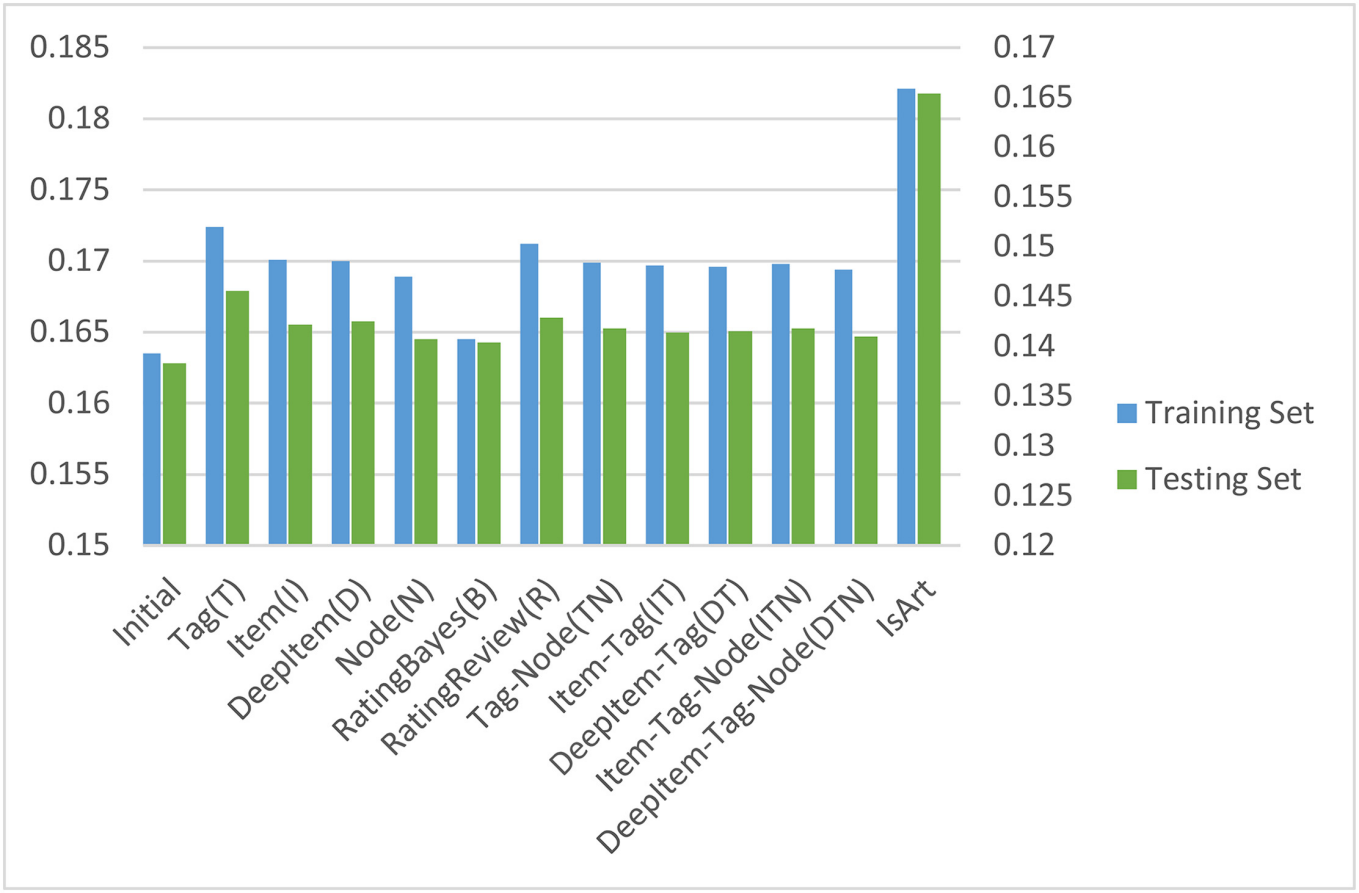
Comparative results (NDCG@10) of promoting book search by reranking with social information (*T*, *N*, *I*, *D*, *R*, *B*, *TN*, *IT*, *DT*, *ITN* and *DTN*), where the initial results are from searching with book contents (without social information) in IsArt.

In this section, we present the performances (NDCG@10) comparison of initial ranking results and all reranking results with social information on INEX Social Book Search 2013 dataset. We select a total of 307 query topics provided by INEX Social Book Search 2011 and 2012 datasets as the training set to construct the proposed reranking model. The best reranking parameters *α* in Eq (5) of all reranking methods are selected according to the best performances on the training set. The initial searching and reranking results on both training and testing sets are shown in Table 6. Specifically, the performance distributions of methods with different types of social information are also presented in Fig 3. The figure gives visualized comparisons of the performances on all reranking methods both on the training set and the testing set.

**Table 6:** Reranking results (NDCG@10) with social information on the training set (INEX Social Book Search 2011 and 2012 datasets) and the testing set (INEX Social Book Search 2013 dataset).

Reranking method	NDCG@10 (training set)	NDCG@10 (testing set)
Initial	0.1635	0.1383
Tag (*T*)	0.1724	0.1456
Item (*I*)	0.1701	0.1422
DeepItem (*D*)	0.1700	0.1425
Node (*N*)	0.1689	0.1407
RatingBayes (*B*)	0.1645	0.1404
RatingReview (*R*)	0.1712	0.1429
Tag-Node (*TN*)	0.1699	0.1418
Item-Tag (*IT*)	0.1697	0.1414
DeepItem-Tag (*DT*)	0.1696	0.1415
Item-Tag-Node (*ITN*)	0.1698	0.1418
DeepItem-Tag-Node (*DTN*)	0.1694	0.1410
IsArt	**0.1821**	**0.1654**

As can be seen from Table 6 and Fig 3, all reranking methods perform well. All reranking results with various social information are more or less better than the corresponding initial searching results on both the training sets and the testing sets. Specifically, the best performance is obtained by the Tag-Rerank method with great improvements of 5.4% and 5.2%, compared with the initial ranking on the training and the testing sets respectively. All these results empirically verify that social information contents (e.g., tags, ratings or reviews) and their combinations can promote book search, therein the reranking methods with recommendation techniques (GCF model) are effective. L2R achieves the best results. In order to know the effectiveness of learning inputs, we list a small subset of the learning parameters to be adjusted in Table 7. As is shown with appropriate parameters, the performances get promoted extensively.

**Table 7:** Results with Random Forest.

Bag	S-rate	F-rate	tc	Tree	NDCG@10
200	1	0.1	11	5	0.1620
200	1	0.2	10	5	0.1597
200	1	0.2	11	4	0.1633
200	1	0.2	11	5	**0.1658**
200	1	0.2	12	5	0.1596
200	1	0.2	13	5	0.1575
200	1	0.3	11	5	0.1611
200	1	0.4	11	5	0.1586
300	1	0.2	11	5	0.1645
300	1	0.3	11	5	0.1605
300	1	0.2	12	5	0.1590
500	1	0.2	11	5	0.1620
500	1	0.3	11	5	0.1597
500	1	0.2	12	5	0.1575

In order to deeply understand the essential reasons, we evaluate these results on different reranking strategies topic by topic. During the evaluation, each reranking result is compared with the initial ranking result to check whether the corresponding reranking strategy is effective, neutral or harmful on the selected topics. The comparison results are shown in Table 8. Despite unavoidable harm when utilizing a single strategy, there are a major part of topics whose performances getting advanced or at least remaining unchanged in evaluation. Taking the case of Tag-Rerank, we pick three topics to show why Tag-Rerank is applicable and how it works. The topic 23796 (listed in Table 9) is a normal one (shown in Table 9) which has only one gold answer (book 517918) with a positive relevant value ranking 13th in initial ranking. However, book 517918 has many common tags with highly-ranked books. Hence, during Tag-Rerank process, this book ranks 5th, which promotes the NDCG@10 value from 0 to 0.4307. Another case occurs in topic 11286, where the gold answers in top 10 of initial results get a better sequence. After reranking, book 374200 with a relevant value 6 and book 364 with a relevant value 2 get a higher ranking position. There are also a lot of topics where no helpful changes took place when reranking. In all the 245 topics where the Tag-Rerank performs neutral, there are 180 topics with 0 evaluated by NDCG@10, which means the initial ranking is so inaccurate that the fine adjustment process like reranking is not enough to be helpful. Among the other 65 topics, the initial results of 62 topics are much better than the mean value with a NDCG@10 value over 0.2. Tag-Rerank performs well, which is probably because the gold answers appear in a relative good position in the initial ranking process and little can be done to be helpful. Moreover, we take topic 1835 (listed in Table 9) as an instance for analyzing how reranking brings harm. The contents show that the topic creator needs an English translation of Homer’s Iliad and most importantly, not all versions are good enough. When checking the gold answers we find that several suggestions have a relevant value with 0, and these suggestions are actually the English versions of Iliad. Our Tag-Rerank method removes one of the gold answers (book 125803) out of top 10 because some other Iliad books have more similar tags with top-ranked books while book 125803 has few tags. When the less relevant books have more similar tags (or other reranking fields) than a relative more relevant book, reranking may bring harm. However as we know, this is not common. Hence, the reranking strategies work by restoring the lost gold answers. However, if the initial ranking performs too good or too bad, the reranking might not helpful. Among the topics for Tag-Rerank, 47 (45.26%) improves over 50% and 90 (87.38%). Consequently, the reranking strategies can improve the initial ranking effectively.

**Table 8:** The distribution of topics (on which whether strategies are effective to the performance of initial ranking measured by NDCG@10) for each reranking strategy (with IsArt as a comparison) on INEX 2013 dataset.

Reranking method	Effective	Neutral	Harmful
Initial	-	386(100%)	-
Tag (*T*)	103(26.69%)	245(63.47%)	38(9.84%)
Item (*I*)	52(13.47%)	309(80.05%)	25(6.48%)
DeepItem (*D*)	52(13.47%)	313(81.09%)	21(5.44%)
Node (*N*)	63(16.32%)	297(76.94%)	26(6.74%)
RatingBayes (*B*)	16(4.15%)	360(93.26%)	10(2.59%)
RatingReview (*R*)	85(22.02%)	265(68.65%)	36(9.32%)
Tag-Node (*TN*)	83(21.50%)	271(70.21%)	32(8.29%)
Item-Tag (*IT*)	39(10.10%)	321(83.16%)	26(6.74%)
DeepItem-Tag (*DT*)	40(10.36%)	328(84.97%)	18(4.66%)
Item-Tag-Node (*ITN*)	39(10.10%)	319(82.64%)	28(7.25%)
DeepItem-Tag-Node (*DTN*)	40(10.36%)	327(84.71%)	19(4.92%)
IsArt	189(48.96%)	185(47.92%)	12 (3.10%)

**Table 9:** Two example topics showing effectiveness or harm during the reranking process.

〈topics〉
〈topic id = “23796”〉
〈query〉colonial-era Maryland Charles Carroll William Paca〈/query〉
〈title〉colonial Maryland suggestions?〈/title〉
〈group〉American History〈/group〉
〈narrative〉 Has anyone got any suggestions for books on colonial-era Maryland? I’m curious to see anything riveting- but would, among other things like to learn more about the role of Annapolis in the revolution and earlyAmerica. Are there any good biographies of Charles Carroll or William Paca?〈/narrative〉
〈/topic〉
〈topic id = “1196”〉
〈query〉iliad english〈/query〉
〈title〉The best English translation of Iliad〈/title〉
〈group〉Homer, the Trojan war, and pre-classical Greece〈/group〉
〈narrative〉 Hello-hello, I have been looking for a good translation of Homer’s Iliad in verse. I have one by Robert Fitzgerald, but I do not find it completely satisfactory. I know there are a lot of English translations of Iliad, and I would love to hear your opinion on which one is the best:)〈/narrative〉
〈/topic〉
〈/topics〉

Another issue that should be of our concern is the difference between these strategies, especially some related strategies and multiple strategies. Different strategies are designed for complementation, and they should produce more different answers. We compare the results of different strategies in pairs (shown in Table 10). From the table we can see that although *Tag—Rerank* alone seems the best, for some specific topics, other mixed models perform better. In order to make use of all the advantages, the linear combination seems not as good as the non-linear combination. Hence, we expect to combine them to the maximum extent with the help of L2R. In summary, IsArt’s unified reranking model with GCF can effectively utilize a variety of diverse social information.

**Table 10:** Comparisons of effectiveness with different pairs of methods.

Method 1	Method 2	topics(Method 1 > Method 2)	topics(Method 1 < Method 2)
Tag	TagNode	89(23.06%)	52(13.47%)
Tag	Item-Tag	87(22.54%)	64(16.58%)
Tag	DeepItem-Tag	97(25.13%)	60(15.54%)
Item	DeepItem	17(4.40%)	19(4.92%)
Item	Item-Tag	31(8.03%)	24(6.22%)
DeepItem	DeepItem-Tag	32(8.29%)	26(6.74%)
Tag-Node	Item-Tag-Node	67(17.36%)	59(15.28%)
Tag-Node	DeepItem-Tag-Node	69(17.88%)	56(14.51%)

### Comparison of IsArt with some other state-of-the-art methods

We extensively perform experiments of IsArt on CLEF/INEX Social Book Search Evaluation datasets of 4 years (from 2011 to 2014), compared with other state-of-the-art methods. In our experiments, the evaluation tool Trec-eval (http://trec.nist.gov/trec_eval/) and the evaluation metric NDCG@10 (Normalized Discounted Cumulative Gain of the top 10 ranking results, *ndcg*_*cut*_10 function in trec-eval) are used for fair comparison, both of which are official in INEX Social Book Search Track. We also present some P@10 (Precision of the top 10 ranking results, *p*_*cut*_10 function in trec-eval), MRR (Mean Reciprocal Rank, *mrr* function in trec-eval), or MAP (Mean Average Precision, *map* function in trec-eval) values according to the official results provided by INEX Social Book Search.

As introduced in Methodology, our ground truth is selected from the suggestions. The evaluation process is to calculate the difference between our results and the ground truth. It is important that some useful suggestions not mentioned in *LT* forum may be missing, which leads the incompleteness of the ground truth. So, *NDCG*@10 scores may be not remarkable. Another issue is that the definition of relevance values changes every year and our evaluation changes with the different relevance values. How we choose the training set and the testing set for each comparative experiment is shown in Table 11. Considering the overlapping topics, we remove the similar or same ones from the training set if they appear in both the training set and the testing set (the number in “()” is the number of removed topics).

**Table 11:** Training and testing sets in experiments.

Group	Training Set	Testing Set
1	74 training topics	211 testing topics
2	2011-211 topics (-50)	2012-96 topics
3	2011-211 topics 2012- 96 topics (-117)	2013-386 topics
4	2011-211 topics 2012- 96 topics (-291) 2013-386 topics	2014-680 topics

INEX 2011 Social Book Search Track announced the task searching books with social information in the INEX evaluations for the first time [43]. The track provides 74 topics for training and 211 topics for testing. The books mentioned in suggestions are set to be relevant (with a relevant value 1) and others are considered as irrelevant (with a relevant value 0). The performance of IsArt and the high-ranked participated teams in the year (2011) are presented in Table 12. top 1 team [27] used different indexes with pseudo-relevance feedback, the 2nd top team [44] and the 3rd top team [13] utilized different query fields to search and fuse these results, and the performances of other teams are not as good. We can see from Table 12 that IsArt performs much better than the participated top teams. Specifically, compared with the best team, IsArt improves the performance (with the measure NDCG@10) by 31.18%.

**Table 12:** Comparison results on INEX 2011 Social Book Search (SBS).

System	NDCG@10	P@10	MRR
IsArt	**0.3423**	**0.2065**	**0.5223**
1st top team of 2011 SBS [27]	0.3101	0.1991	0.4811
2nd top team of 2011 SBS [44]	0.2991	0.1910	0.4731
3rd top team of 2011 SBS [13]	0.2913	0.1858	0.4661

INEX 2012 Social Book Search Track uses the personal catalogue of topic creators to distinguish among forum suggestions. These suggestions include 1) suggestions which already are in their catalogues are given with the relevant value 0 because they seems not helpful; 2) suggestions that are decided and added to the user’s catalogue, and satisfy the requests of topic creators and its relevant value is defined to be 4; and 3) suggestions which are given a medium value “1” The relevance values for suggested books are first distinguished based on catalogues. The training set is the official database of INEX 2011, which contains 211 topics, while the testing set contains 96 topics.

IsArt’s performance and the high-ranked results of top participated teams in the year (2012) [1] are presented in Table 13. The 1st top team (Bogers and Larsen’s team) fused ratings, tags and authors for book similarity reranking [28]. The 2nd top team (Huurdeman et al.’s team) used a Bayesian rating method with Collaborative Filtering [45]. The 3rd top team (Bonnefoy et al.’s team) used some social information with a sequential dependence model [25]. As can be seen from Table 13, the proposed IsArt performs much better than the participated top teams.

**Table 13:** Comparison results on INEX 2012 Social Book Search (SBS).

System	NDCG@10	P@10	MRR
IsArt	**0.1923**	**0.1497**	**0.4032**
1st top team of 2012 SBS [16]	0.1492	0.1198	0.3069
2nd top team of 2012 SBS [45]	0.1460	0.1380	0.370
3rd top team of 2012 SBS [14]	0.1339	0.1260	0.3410

In INEX 2013 Social Book Search Track, the field 〈query〉 is firstly used, and the relevance values are redefined because several additional factors are considered. Single judgement/multiple judgements, reading/not reading judgements, the number of positive/neutral/negative judgements are combined together to build a decision tree [2]. The suggestions are re-collected and set 7 different levels of relevant values (0/1/2/3/4/6/8). The training set contains more than 300 topics (211 + 96, including some duplicated topics) and the testing set contains 386 topics. The performance of IsArt and the high-ranked participated teams of the 2013 evaluation are shown in Table 14. Bogers and Larsen [46] (the top 1 team) improved their system of the previous year [28] by expanding the XML document. The second group from University of Amsterdam [2] tried to choose different fields of queries to improve their Bayesian rating method [45]. The 3rd top team [15] used free text queries with a Krovetz stemmer and stop-words removed index [2]. These results also verify that IsArt performs the best as compared to all other evaluated systems.

**Table 14:** Comparison on INEX 2013 Social Book Search.

System	NDCG@10	P@10	MRR
IsArt	**0.1654**	**0.0821**	**0.2465**
1st top team of 2013 SBS [46]	0.1361	0.0653	0.2286
2nd top team of 2013 SBS [40]	0.1331	0.0771	0.2342
3rd top team of 2013 SBS [15]	0.1150	0.0479	0.1839

In INEX 2014 Social Book Search Track, we participated in the Suggestion Task with IsArt and won the evaluation [47]. According to the public evaluation results (https://inex.mmci.uni-saarland.de/tracks/books/INEX14_SBS_resultsv2.jsp), the NDCG@10 results of 18 runs, among all 40 submitted runs, are over 0.10. Some evaluation results are shown in Table 15. The results in “()” are the original ones by IsArt. Noticing that the results are slightly lower than the performance of Hafsi’s methods, we re-studied Hafsi’s methods and found that they remove the stopwords of original documents, While in our approach we only filter out the stopwords of the queries, rather than the documents. To be fair, we chose to implement the same strategy and then compare our work with Hafsi’s “BM25F” model. The results in bold are those by using IsArt. The method of the 2nd top team modifies the BM25 model [50] and proposed “BM25F” model on the title, mediated query and narrative fields, with the parameters optimized for the narrative field, while the 3rd top team’s approaches were based on Sequential Dependence Model [51] with pseudo-relevance feedback and query expansion. Methods from the other teams with indexing strategies or query fields selection did not behave as good as previous years. The 4th run has no workshop associated, so we do not know the corresponding methods. Again, IsArt has a better performance compared with other top ranked participated systems. We can easily see that IsArt has a greater performance than Hafsi’s BM25F.

**Table 15:** Participating results on INEX 2014 Social Book Search Suggestion Task.

System	NDCG@10	MRR	MAP
IsArt (Top 1 team of 2014 SBS) [47]	**0.165** (0.141)	**0.298** (0.254)	**0.106** (0.101)
2nd top team of 2014 SBS [48]	0.142	0.275	0.107
3rd top team of 2014 SBS [49]	0.128	0.236	0.101
4th top team of 2014 SBS (no workshop paper)	0.127	0.239	0.097

### Statistical Significance Testing and Results Analysis

In order to report effect sizes and confidence intervals more informatively, the statistical significant testing has been conducted according to Tetsuya Sakai’s 2014 SIGIR Forum paper [52].

As shown in Tables 10–13, the submitted runs from Bogers, Koolen, Bellot and Hafsi show great effectiveness and robustness. We implement their approaches and compare the runs with our results. According to the evaluation results, the methods from Bogers, Koolen, Bellot are basically replicated while the performances of the method from Hafsi is slightly lower than the statistics in Table 15. Hence, we first compare our results with each one on the selected datasets (with 386 topics in SBS 2013). According to the two-sided paired t-test experiments for the difference in the mean value $\bar{d}=0.0102$ (with the unbiased estimation of the population variance V = 0.0066), our system statistically significantly outperforms Bogers’ system (*t*(386) = 2.4667, *p* < 0.0141, *ES*_*pairedt*_ = 0.1256, 95% CI [0.0021,0.0183]). The other three pairs of comparison shows similar results and the exact result values are shown in Table 16.

**Table 16:** Two sided paired t-test results on our system with 4 baselines.

Baseline	$\bar{d}$	t	p(<)	*ES*_*pairedt*_	95% CI
From Bogers	0.0102	2.4667	0.0141	0.1256	[0.0021,0.0183]
From Koolen	0.0102	2.3246	0.0206	0.1183	[0.0016,0.0188]
From Bellot	0.0128	2.6812	0.0077	0.1364	[0.0034,0.0221]
From Hafsi	0.0077	2.3214	0.0208	0.1181	[0.0012,0.0142]

In order to analyze our proposed IsArt technology, we also utilize a two-way ANOVA test to accomplish our statistical significance testing on different components of our system, including an initial ranking system, 11 reranking systems and a Random-Forest system. Table 17 shows the results of a two-way ANOVA (without replication) experiments which are conducted for the comparison of such m = 13 systems with n = 386 topics. The system is statistically significant (*F*(12,4620) = 1.699, *p* < 0.00602)^5018^. The population effect size and the partial population effect size for the ANOVA can be estimated from Table 17 as *ω*^2^ = *ϕ*_*A*_(*V*_*A*_−*V*_*E*_)/(*S*_*T*_ + *S*_*B*_) = 6.659 * 10^−5^ and $\omega_{p}^{2}=\phi_{A}\left(V_{A}-V_{E}\right)/\left(S_{A}+\left(n-\phi_{A}\right)V_{E}\right)=0.0257)$.

**Table 17:** Two-way ANOVA (without replication) on components of our systems.

	Sum of squares	Degree of freedom	Mean Squares	*F*_0_
Between-system	*S*_*A*_ = 0.0318	*ϕ*_*A*_ = 12	*V*_*A*_ = 0.0029	1.699
Between-topic	*S*_*B*_ = 190.55	*ϕ*_*B*_ = 385	*V*_*B*_ = 0.6548	384.511
Within	*S*_*E*_ = 5.4511	*ϕ*_*E*_ = 4620	*V*_*E*_ = 0.0017	-

**Fig 4:**
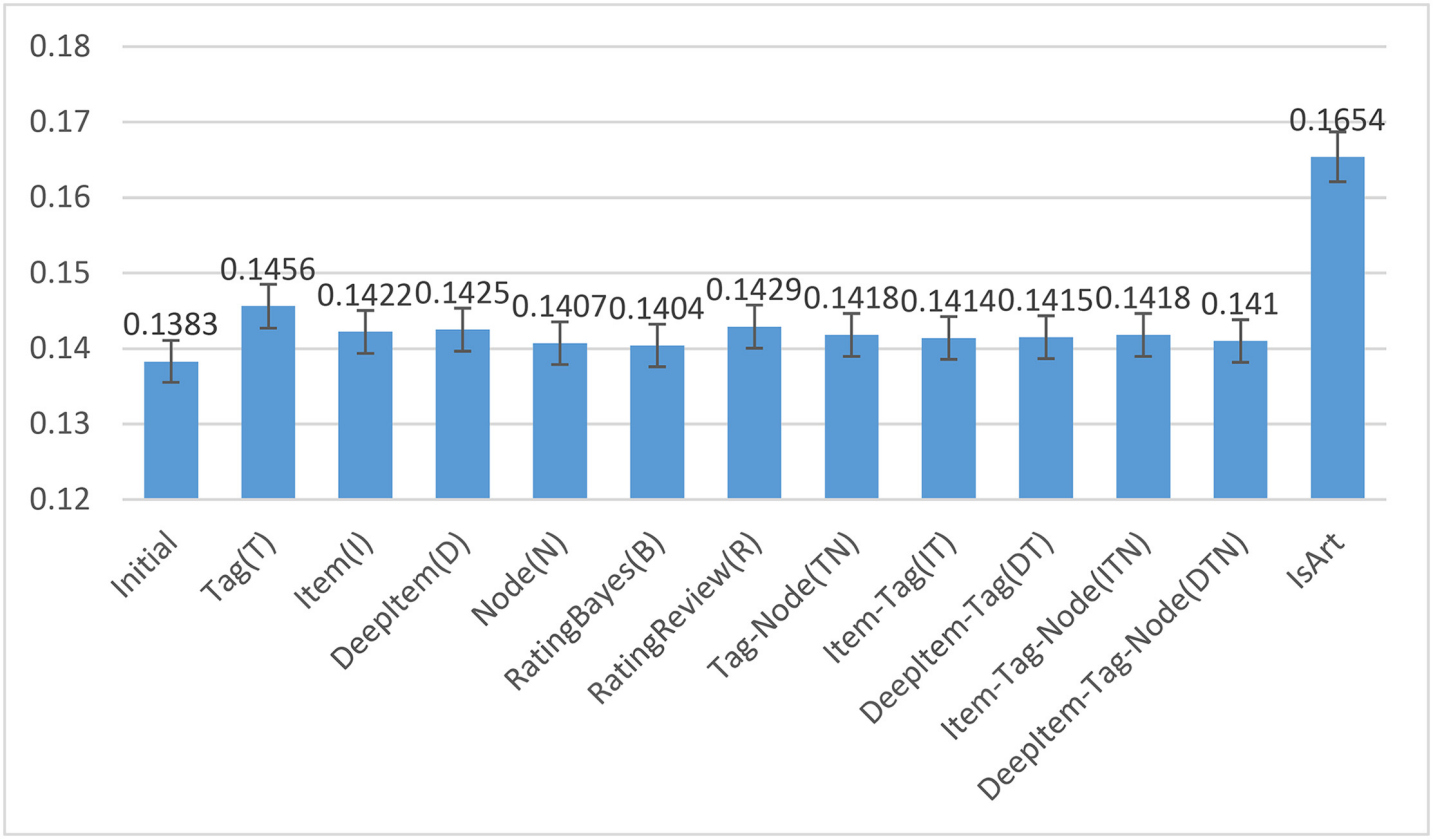
The NDCG@10 performances of initial ranking, 11 reranking and IsArt with 95% CIs using the same VE from Table 17.

A randomised Tukey HSD test shows that except RatingBayes-Rerank and initial ranking, the difference between each reranking system and the initial system, as well as the difference between IsArt and each reranking system are statistically significant (*p* < 0.05). Fig 4 shows the mean performances of the 13 systems with 95% CIs using the same VE from Table 17.

Through the above comparisons and the statistical significance testing, we can draw the conclusion that our system with IsArt outperforms other systems. There are several reasons leading to the improvements. Firstly, IsArt is designed with better extendible and versatile. IsArt is a framework to improve the performance through optimizing the initial ranking results. Hence, any method that improves the initial ranking can be utilized in the initial ranking process. Similarly, IsArt is designed for social information, so any types of social information, which can represent the content of books to some extent, can be transformed into one of the representations mentioned in the GCF model. Furthermore, other than social information, some controlled metadata, for instance, *browsenode*, reflects some kind of information of the books, can be also applied to the framework. Secondly, it is about the complexity of the track. As mentioned in the Methodology section, many aspects should be concerned for Social Book Search. The ground truth is extracted from the suggestions, which means some content-related books not mentioned in any suggestions are not popular because few people know these books. The definition of the relevance values indicates that although some books are relevant to queries, they may not be useful or helpful because they cannot satisfy the topic creators’ preferences or they are already known by the topic creators. However in a common retrieval system, neither the popularity nor the preferences can be reflected by a conventional model. These are usually measured in the recommendation models, such as Collaborative Filtering or Content-based Filtering. IsArt investigates the similarity of the results, which is similar to the core procedure in Content-based Filtering.

Reuter [53] identified 7 general categories of relevance aspects for book search, i.e., Metadata, Accessibility, Content, Engagement, Novelty, Familiarity and Socio-Cultural.

**Metadata**. Books with a certain title or by a certain author, editor, illustrator, publisher, in a particular format, or written.

**Accessibility**. The language, length or level of difficulty of a book.

**Content**. Aspects such as topic, plot, genre, style or comprehensiveness of a book.

**Engagement**. Books that fit a particular mood or interest, or books that are considered high quality or provide a particular reading experience.

**Novelty**. Books with novel content for the reader, books that are unusual.

**Familiarity**. Similar to known books or related to previous experience.

**Socio-Cultural**. Books related the user’s socio-cultural background or values, books that are popular or obscure.

According to Social Book Search organizers [2, 32], the topics only involve Accessibility, Content, Engagement, Metadata(very few) and an added category, **Known-Item**, which means the description of known books to identify the title and/or author, or publishing year or period. For the Accessibility, we do not think IsArt can work better. Moreover, for the Metadata, IsArt only plays the role of ad-hoc reranking, with minor effectiveness. However for the Content, Engagement and Familiarity, investigating the relationship among books helps IsArt to achieve a better results than other state-of-the-art models (shown in Table 18). To sum up, IsArt provides an effective and robust framework to promote Social Book Search.

**Table 18:** The analysis of aspects of topics (number of topics IsArt better than Initial ranking).

Aspects	Total	IsArt> Initial
access	106	15(14.1%)
content	523	294(56.2%)
cultural	108	37(34.3%)
engagement	154	79(51.2%)
familiarity	261	152(58.2%)
known-item	97	30(30.9%)
metadata	177	42(23.7%)
novelty	29	10(34.5%)

## Conclusion and Discussion

Here we develop a novel book search system, IsArt, which provides a generic framework for searching books by utilizing a wide diversity of rich social information to promote book search effectiveness on the Web. In this generic framework, a search engine first ranks books with book contents. Through searching, a ranking list of books is available for each topic. Then, a reranking model re-ranks books with various social contents (e.g., ratings, tags and reviews) through a new recommendation model, Generalized Content-based Filtering (GCF). From this aspect, IsArt embeds a recommendation system for searching and finding books. Furthermore, a learning-to-rank (L2R) technique with Random Forests adaptively combines various and diverse reranking results. By using L2R, IsArt (the whole system) becomes an integrated search and recommendation technology with supervised learning for searching books. IsArt is experimented and verified on INEX Social Book Search Evaluation datasets of all 4 years (from 2011 to 2014). For searching books on this collection of 2.8 million books with a massive number of user-generated social contents, IsArt outperforms all other state-of-the-art systems.

How does social information help search and find books? Extensive experiments of the reranking model with GCF in IsArt on INEX Social Book Search Track verify that social information can promote book search. Generally speaking, reviews contain a large amount of information related to contents, tags are the keywords of a book which generalize the books sweepingly, ratings stand for the concerns of readers which help to rule out unpopular books, and similar products show the books frequently bought together which indicate that the similar products of a relevant book may be useful too. Consequently, searching books with social information, e.g., IsArt, can effectively improve the searching and finding results.

Another challenge in social book search is how to understand the semantic query, a complicated but hot research topic in information retrieval and recommendation systems. IsArt is an effective way to meet this challenge, however, to solve this problem, more techniques are needed to be further investigated. One direction of future work is to construct the logical structure of query words with semantic parsing and corpus knowledge.

Finally, IsArt and the proposed GCF model can be easily applied to most product search systems like Social Book Search, and the only difference is the way of feature extraction for various structures of the product descriptions. As a result, another future work is to investigate this GCF model and then IsArt for applications in general searching and social searching systems for searching and navigating products with rich social information on the Web (e.g., Amazon.com or Taobao.com).
